# Cellular adaptation to xenobiotics: Interplay between xenosensors, reactive oxygen species and FOXO transcription factors

**DOI:** 10.1016/j.redox.2017.07.015

**Published:** 2017-08-03

**Authors:** Lars-Oliver Klotz, Holger Steinbrenner

**Affiliations:** Institute of Nutrition, Department of Nutrigenomics, Friedrich-Schiller-Universität Jena, D-07743 Jena, Germany

**Keywords:** AhR, arylhydrocarbon receptor, AIP, AhR interacting protein, ARE, antioxidant response element, ARNT, AhR nuclear translocator, bHLH, basic helix-loop-helix (DNA binding and dimerisation domain), CAR, constitutive androstane receptor, CBP, CREB-binding protein, *C. elegans*, *Caenorhabditis elegans*, CYP, cytochrome P450, DAF-16, abnormal dauer formation 16, DBE, DAF-16 binding element (a FOXO-responsive DNA element), DEM, diethyl maleate, EpRE, electrophile response element, FOXO, forkhead box class O, G6Pase, glucose 6-phosphatase, GCS, γ-glutamylcysteine synthetase, GST, glutathione S-transferase, HNE, 4-hydroxynonenal, HNF4α, hepatocyte nuclear factor 4α, Keap-1, Kelch-like ECH-associated protein 1), NQO1, NAD(P)H:quinone oxidoreductase-1, NOX4, NADPH oxidase 4, Nrf2, nuclear factor erythroid 2-related factor 2, also known as nuclear factor (erythroid-derived-2)-like 2, PAH, polycyclic aromatic hydrocarbons, PBRE, phenobarbital response element, PEPCK, phosphoenolpyruvate carboxykinase, PGC, PPARγ coactivator, PPAR, peroxisome proliferator-activated receptor, PI3K, phosphoinositide 3‘-kinase, PPRE, PPAR response element, PXR, pregnane xenobiotic receptor, PXRE, PXR response element, ROS, reactive oxygen species, RXR, retinoid X-receptor, SKN-1, skinhead 1, SOD, superoxide dismutase, SULT, sulfotransferase, TCDD, 2,3,7,8-tetrachlorodibenzo-p-dioxin, UGT, UDP-glucuronosyl transferase, Xb, xenobiotic, XRE, xenobiotic response element, Xenobiotic metabolism, Biotransformation of xenobiotics, Forkhead box transcription factors, Redox regulation

## Abstract

Cells adapt to an exposure to xenobiotics by upregulating the biosynthesis of proteins involved in xenobiotic metabolism. This is achieved largely via activation of cellular xenosensors that modulate gene expression. Biotransformation of xenobiotics frequently comes with the generation of reactive oxygen species (ROS). ROS, in turn, are known modulators of signal transduction processes. FOXO (forkhead box, class O) transcription factors are among the proteins deeply involved in the cellular response to stress, including oxidative stress elicited by the formation of ROS. On the one hand, FOXO activity is modulated by ROS, while on the other, FOXO target genes include many that encode antioxidant proteins – thereby establishing a regulatory circuit. Here, the role of ROS and of FOXOs in the regulation of xenosensor transcriptional activities will be discussed. Constitutive androstane receptor (CAR), pregnane X receptor (PXR), peroxisome proliferator-activated receptors (PPARs), arylhydrocarbon receptor (AhR) and nuclear factor erythroid 2-related factor 2 (Nrf2) all interact with FOXOs and/or ROS. The two latter not only fine-tune the activities of xenosensors but also mediate interactions between them. As a consequence, the emerging picture of an interplay between xenosensors, ROS and FOXO transcription factors suggests a modulatory role of ROS and FOXOs in the cellular adaptive response to xenobiotics.

## Highlights

• Exposure of cells to xenobiotics may trigger formation of reactive oxygen species.
• Xenosensors respond to xenobiotics by upregulation of xenobiotic metabolism.
• FOXO transcription factors modulate the activities of several xenosensors.
• ROS affect FOXO activity, and FOXO target genes include antioxidant proteins.
• FOXOs bridge xenobiotic-induced ROS generation and xenosensor regulation.

## Graphical abstract

**Figure:**
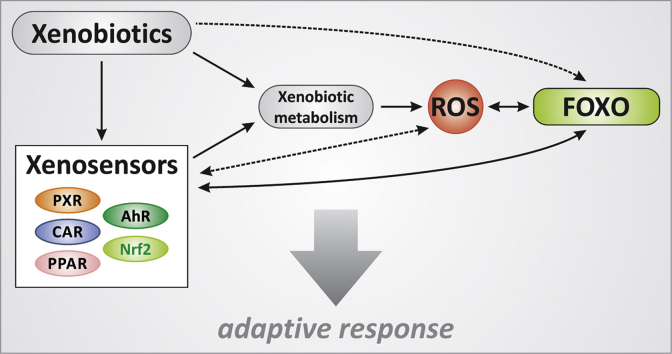

## 1 Introduction

Exposure of mammalian cells to xenobiotics – i.e., compounds that are “foreign” to the organism of interest, such as (environmental) toxins, metal ions, drugs, phytochemicals – elicits responses ranging from signaling and adaptation to cell death. Cells are equipped with enzymatic means of metabolizing xenobiotics for the purpose of eliminating these compounds. Xenobiotic metabolism and biotransformation occurs in stages; for example, in the case of hydrophobic xenobiotics, these are devoted to generating functional groups in these compounds (phase I) that serve as docking sites for hydrophilic compounds they are coupled with (phase II). These transformations then allow for transport and excretion (phase III) of xenobiotic metabolites.

Cells react to an exposure to xenobiotics by upregulating the formation of the xenobiotic metabolism machinery, i.e. of proteins involved in the above-mentioned phases of biotransformation. This adaptive cellular response is largely due to the interaction of xenobiotics with signaling cascades and transcriptional regulators, i.e. “xenosensors”. Cellular structures that are targeted by xenobiotics, triggering a cellular response, may of course be considered as xenobiotic sensors, i.e. xenosensors, in a very broad sense. However, the actual term rather refers to proteins less “accidentally” interacting with their ligands. Here, we will focus on xenosensors in the latter sense, i.e. xenobiotic-sensitive transcriptional regulators.

**Fig. 1:**
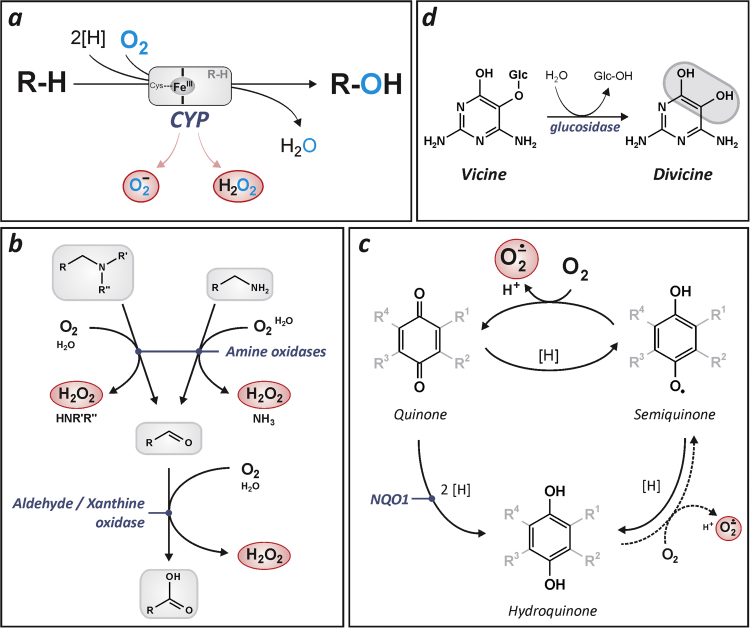
Xenobiotics and the formation of reactive oxygen species. Reactive oxygen species are generated during xenobiotic metabolism through oxidation, reduction as well as hydrolytic processes. (**a**) Cytochrome P450 (CYP) monooxygenase activity requires both electrons and the activation of molecular oxygen. Accordingly, oxygen reduction products, such as superoxide and hydrogen peroxide may leak out of the enzyme complex – at least in vitro [56]; it is not entirely clear in how far this contributes to xenobiotic toxicities in vivo [3]. CYPs may also contribute to the generation of reactive oxygen species by catalyzing the formation of products that may then undergo oxidative processes. (**b**) Certain amines and aldehydes may be metabolized by oxidases that generate hydrogen peroxide. (**c**) Redox cycling: reduction of certain compounds may lead to the formation of products that are reoxidized by molecular oxygen, which is present in significant concentrations in cells and tissues. Molecular oxygen, in that same process, may be reduced to superoxide, which will undergo (spontaneous or enzyme-catalyzed) dismutation to generate hydrogen peroxide. Here, the endogenous reduction of quinones to semiquinones and hydroquinones is shown. NAD(P)H:quinone oxidoreductase-1 (NQO1) catalyses the two-electron reduction of quinone substrates to generate hydroquinones that may then undergo phase II metabolism to form hydrophilic adducts. NQO1 is thus an antioxidant enzyme, provided the generated hydroquinone is passed on to phase II metabolism prior to its reoxidation by molecular oxygen [57]. (**d**) Even hydrolysis may contribute to the generation of ROS: vicine (found in fava beans) may be hydrolyzed after ingestion (likely by action of intestinal microbiota) to generate its aglycon, divicine [58]. The latter is an o-hydroquinone, which may, in turn, undergo redox cycling [59].

Biotransformation comes with the generation of reactive oxygen species (ROS) through a multitude of reactions both directly releasing ROS as part of the respective reaction and indirectly as a consequence of the products generated by transformation of a xenobiotic [1], [2], [3] (Fig. 1). The generation of ROS is a natural consequence of biotransformation using serial redox reactions and exploiting the presence of oxygen. Electron transfer to molecular oxygen will result in the generation of superoxide and derivatives thereof, such as hydrogen peroxide. These ROS (this generic term will be used here when referring to superoxide and/or hydrogen peroxide) are known modulators of cellular signaling processes by interfering with signaling cascades at several levels, including at the level of transcriptional regulators. Moreover, cells are now known to exploit the transient formation of superoxide/hydrogen peroxide as vital components of signaling cascades, including growth factor-dependent signaling.

In summary, xenobiotics trigger xenosensor-dependent adaptation of xenobiotic metabolism, and in parallel, through xenobiotic metabolism, contribute to the generation of ROS and to ROS-dependent regulation of cellular signaling events.

Several transcription factors are now known to be redox-regulated and affected by the generation of ROS (see [4] for a comprehensive review). Here, we will focus on a group of factors known not only to be regulated by ROS but to also control cellular stress response and antioxidant defense: Forkhead Box, class O (FOXO) transcription factors.

The purpose of this article is to provide a brief overview on the interplay between xenosensors, ROS and FOXO transcription factors in regulating the cellular response to an exposure to xenobiotics. Following a discussion of the modulation of FOXO signaling by ROS, we will provide examples of xenosensors directly targeted by xenobiotics (CAR, PXR, PPARs, AhR and Nrf2) and delineate their relation with ROS and FOXOs.

## 2 Modulation of FOXO transcriptional activity: “ROS” and “non-ROS” routes

Metabolism of xenobiotics may cause the generation of ROS (most frequently, superoxide and hydrogen peroxide, see Fig. 1), which, in turn, were demonstrated to affect the activity of FOXO transcription factors (for a recent summary, see [5]; in short, both activation and inactivation of FOXOs may be elicited by ROS, depending on location, timing and extent of their formation). Four FOXO isoforms (FOXOs 1, 3, 4, 6) exist in humans that are ubiquitously expressed (albeit with varying expression levels in different tissues); currently, most data in the body of literature on FOXOs is on isoforms FOXO1, FOXO3 and FOXO4. FOXO transcription factors not only regulate the expression of genes encoding proteins involved in antioxidant defense [5], but also regulate the formation of enzymes involved in fuel metabolism [6] or the regulation of cellular proliferation and cell death [7], among many others.

### 2.1 Regulation of FOXOs by ROS

**Fig. 2:**
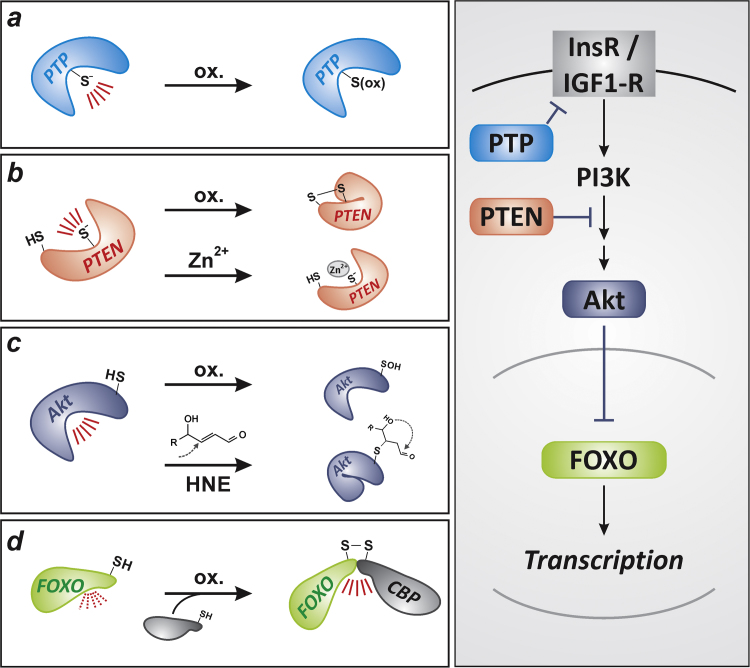
“ROS” and “non-ROS” routes for modulation of FOXO activity along the insulin receptor/IGF-1 receptor dependent signaling cascade. Right panel: Insulin (or insulin-like growth factor, IGF1), via stimulation of insulin (or IGF1) receptor (InsR, or IGF1-R), via phosphoinositide 3′-kinase (PI3K)-induced generation of 3′-phosphorylated phosphoinositides and via the subsequent activation of the serine/threonine kinase Akt, causes inactivation of FOXO transcription factors. This cascade is controlled by protein tyrosine phosphatases (PTPs) such as PTP1B that dephosphorylate and inactivate the insulin receptor. Moreover, the lipid phosphatase, PTEN (phosphatase and tensin homolog) attenuates PI3K signaling by catalyzing the 3′-dephosphorylation of phosphoinositides. (**a**) Most known PTP harbor an oxidation-sensitive (low-pK_a_) cysteine at their active site and may therefore be inactivated by oxidants such as H_2_O_2_, peroxynitrite or singlet oxygen [60]. This inactivation may be reversible, depending on the extent of oxidation [61]. (**b**) Similar to PTPs, PTEN may be inactivated by oxidation, e.g. upon exposure to H_2_O_2_, which yields a disulfide [62]; PTEN inactivation may also occur in a “non-ROS” fashion, e.g. through interaction with metal ions such as Zn^2+^[63]. (**c**) Akt oxidation, e.g. to form sulfenylated Akt [64], [65], and reaction with electrophiles, such as a Michael addition of the lipid peroxidation product 4-hydroxynonenal (HNE) to a susceptible cysteine residue (i.e. HNEylation of Akt), result in Akt inhibition [11]; (**d**) FOXO transcription factors may be regulated by hydrogen peroxide-induced covalent binding to coregulators such as CBP, which can both affect acetylation status of FOXOs as well as of histones near the transcription start site [9], [10].

ROS-dependent FOXO modulation occurs at several stages: FOXO protein levels, for example, are regulated posttranscriptionally by redox-sensitive RNA binding proteins or redox-regulated microRNAs (for a recent review, see [8]). FOXO activity, in turn, is modulated by upstream signaling cascades that affect FOXO subcellular localization, DNA binding and transactivation activity. The most prominent of these cascades is the signaling cascade emanating from insulin receptor or insulin-like growth factor-1 (IGF-1) receptor which, via phosphoinositide 3-kinase (PI3K) and the serine/threonine kinase Akt, causes phosphorylation and inactivation as well as nuclear exclusion of FOXOs [6]. As described in Fig. 2, this cascade is affected by conditions causing the generation of ROS. Moreover, FOXOs themselves were also demonstrated to interact with coregulators, such as CREB-binding protein (CBP), via formation of intermolecular disulfides if exposed to hydrogen peroxide [9], [10]. As several FOXO target genes encode proteins involved in antioxidant defense, those may in turn decrease intracellular ROS levels that would otherwise modulate the activation of FOXOs. Importantly, oxidative processes elicited by ROS may generate reaction products that affect cellular signaling cascades. One such example is 4-hydroxynonenal (HNE), which is a lipid peroxidation product and may interact with Akt [11] (see Fig. 2**c**).

### 2.2 ROS-independent regulation of FOXOs by xenobiotics

Several xenobiotic compounds may affect FOXO signaling also independently of the formation of ROS. Here, we will briefly analyze the effects of metal (Cu, Zn) ions that – albeit of physiological relevance – may also trigger undesired effects, of metalloids such as arsenic, as well as of naphthoquinones of various origins. These compounds share an affinity for susceptible thiol moieties on proteins.

Whereas oxidation of proteins by ROS may be reversible (e.g., thiol oxidation to disulfide or to sulfenic acid may be reversed intracellularly, in part through the action of enzymes of the redoxin family [12]), the non-oxidative interaction with xenobiotics may or may not be. For example, metal ions can interfere with enzyme activities (such as Zn ions with PTEN, see Fig. 2**b**), but binding is noncovalent and usually reversible; in contrast, alkylation (such as a Michael addition of electrophiles to thiols or to amino groups of proteins) usually yields irreversibly modified proteins (e.g., Fig. 2**c**; the endogenous lipid peroxidation product HNE is given as a representative of such electrophilic compounds).

Similar to Zn ions, Cu ions (and, to some extent, also Ni ions [13]) were demonstrated to stimulate PI3K/Akt signaling and to result in FOXO inactivation (phosphorylation and nuclear exclusion) [14]. Another “thiophilic” compound, arsenite, was also demonstrated to modulate the insulin signaling cascade and FOXO-dependent gene expression in HaCaT human keratinocytes [15] and HepG2 human hepatoma cells [16]. Both copper- and arsenite-induced modulation of FOXO signaling appears to be independent of the insulin receptor [16], [17]. Cu-induced effects were also suggested to be independent of the generation of ROS [18]. This is in line with the stimulation of other receptor tyrosine kinase pathways by certain xenobiotics, such as the stimulation of the EGF receptor by naphthoquinones [19]. 1,4-Naphthoquinones – through redox cycling (Fig. 1**c**) – induced oxidative DNA damage and the oxidation of glutathione in human keratinocytes. Nevertheless, the concomitantly induced strong stimulation of the EGF receptor tyrosine kinase was attenuated by a maximum of 20% in the presence of a Mn porphyrin that has both superoxide dismutase and catalase-like activity. This implies that the majority of the effects of these naphthoquinones on signaling was independent of the formation of superoxide or hydrogen peroxide [20].

## 3 Interactions between FOXOs and xenosensors

**Fig. 3:**
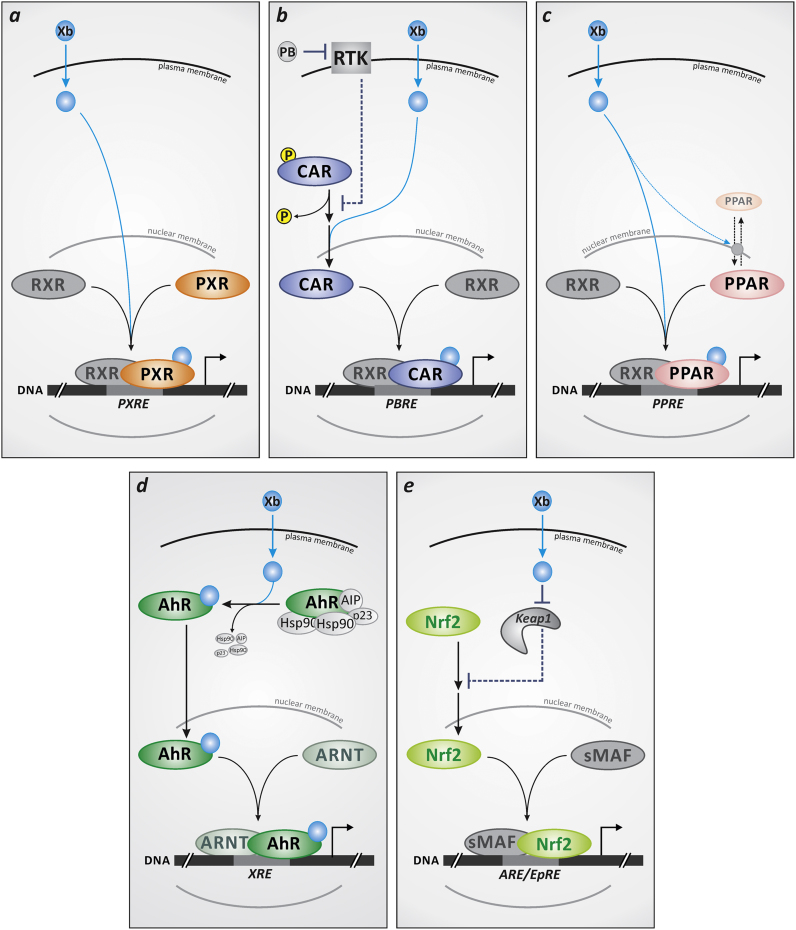
Xenosensor activation by xenobiotics (Xb): simplified schematic representation. (**a,b**) Human pregnane X receptor (PXR) as well as retinoid X receptor (RXR) alpha appear to occur predominantly in the nucleus in unstimulated cultured human cells [66], [67], whereas constitutive androstane receptor (CAR) occurs mostly cytosolic [67]. Upon stimulation by exposure to xenobiotics (i.e. upon ligand binding), both PXR and CAR stimulate the expression of target genes by binding to their respective response elements (PXRE, PBRE) as heterodimers with RXRα. Nuclear translocation of CAR is regulated by its (de)phosphorylation: protein kinase C-dependent phosphorylation induces its cytoplasmic retention [68], which is further supported by receptor tyrosine kinase (RTK)-dependent effects. For example, epidermal growth factor (EGF) induces, via activation of ERK, the formation of a CAR homodimer which prevents CAR dephosphorylation and thus helps retain it in the cytoplasm [69]. CAR ligands bind to the monomer, and by shifting the dimer/monomer equilibrium accordingly support CAR dephosphorylation, followed by nuclear translocation [69]. Another type of CAR activator, phenobarbital (PB) stimulates CAR indirectly, e.g. by interfering with RTK signaling: both EGF and insulin binding to their cognate receptors are attenuated in the presence of PB [30], [31], thus counteracting the CAR inactivation elicited by EGF or insulin. (**c**) Like PXR and CAR, peroxisome proliferator-activated receptors (PPARs) form heterodimers with RXR. RXR/PPAR heterodimers bind to PPRE (peroxisome proliferator response element) sites to stimulate transcription of target genes. Although PPARs were described as predominantly residing in the nucleus, certain stimuli were reported to elicit nuclear exclusion [70]. Moreover, nucleocytoplasmic shuttling and subcellular localization of PPARs was shown to be affected by PPAR ligands [36]. (**d**) Aryl hydrocarbon receptor (AhR) is retained in the cytoplasm in a complex containing heat shock protein (hsp) 90 as well as AhR inhibitory protein (AIP) and p23. Ligand binding releases AhR from this complex, followed by its nuclear translocation. AhR forms a heterodimer with AhR nuclear translocator (ARNT) inside the nucleus; the dimer then binds to xenobiotic response elements to stimulate transcription of target genes [44]. (**e**) Stimulation of Nrf2 transcriptional activity by xenobiotics is through interaction of xenobiotics with Keap-1, releasing Nrf2 from a complex with Keap-1 (see legend to Fig. 5). Following nuclear translocation, Nrf2 forms heterodimers, e.g. with small musculoaponeurotic fibrosarcoma (sMaf) proteins, that bind to antioxidant response elements (ARE) (also termed electrophile response element, EpRE) and regulate transcription [49].

Here, we will briefly introduce xenosensors that FOXO proteins were demonstrated to interact with, discuss the nature of this interaction and the role of ROS therein. In this regard, Fig. 3 presents an overview on the modes of activation of these xenosensors by their ligands (e.g., xenobiotics).

### 3.1 CAR/PXR

Constitutive androstane receptor (CAR) and pregnane xenobiotic receptor (PXR) are RXR heterodimer forming members of the nuclear receptor family (Fig. 3**a/b**) and are widely regarded as the major xenosensors as they interact with a multitude of ligands and drive the transcription of genes encoding major phase I/II enzymes, including CYP2C and CYP3A monooxygenases, which were estimated to catalyse approx. 60% of all cytochrome P450 (CYP)-mediated drug oxidations [21], as well as UDP-glucuronosyl transferases (UGTs), sulfotransferases (SULTs) and others. As with their target genes, there is an overlap between the two receptors also with respect to their activators, although ligands selective for CAR (such as phenytoin) as well as PXR (such as rifampicin and hyperforin) were identified [22], [23], [24]. For a list of CAR and PXR activators as well as target genes, see [25].

Both CAR and PXR were demonstrated to interact with FOXO1 in HepG2 human hepatoma cells, and FOXO1 was then identified as a coactivator of CAR and PXR in these cells [26]. As expected for a FOXO-dependent effect, this activity was attenuated by insulin via PI3K and Akt [26]. Interestingly, while FOXO1 coactivates CAR and PXR, the two latter (as CAR/RXR or PXR/RXR heterodimers) were shown to act as corepressors of FOXO activity on its DNA binding elements [26]. This interaction was demonstrated to result in a CAR-induced attenuation of FOXO target gene expression, such as of genes coding for gluconeogenesis enzymes, phosphoenolpyruvate carboxykinase (PEPCK) and glucose 6-phosphatase (G6Pase), or the cyclin-dependent kinase inhibitor, p21, and also FOXO1 itself [27]. CAR-mediated suppression of FOXO1 (along with hepatocyte nuclear factor 4α, HNF4α) was suggested to be responsible for the anti-gluconeogenesis effects of certain alkaloids [28]. Similarly, PXR activation was shown to affect gluconeogenesis not only through FOXO1 inhibition but also through competition for PPARγ coactivator (PGC) 1α, a known coregulator of FOXOs as well as other transcriptional regulators, such as HNF4α (for review, see [29]).

An interaction between insulin signaling and CAR was shown in (murine and human) hepatocytes as insulin not only causes the above inactivation of FOXOs (and thus a loss of coactivation of CAR) but also attenuates CAR activation [30] – likely at the level of preventing its dephosphorylation, as seen also with EGF [31]. Therefore, a direct link exists between energy and xenobiotic metabolism through FOXO/CAR interaction and through the effect of insulin on CAR activation. Based on these findings, two types of CAR activators can be distinguished – those acting as CAR ligands (e.g., CITCO) and those stimulating CAR in an indirect fashion, via control of CAR dephosphorylation, such as phenobarbital. The latter was demonstrated to interfere with binding of EGF or insulin to their respective receptors [30], [31], hence attenuating CAR inactivation.

Regarding the role of ROS in fine-tuning CAR/PXR signaling, one can reasonably assume an interaction with these nuclear receptors through the known capability of ROS to modulate FOXO signaling. A recent study suggests that the stimulation of CAR-dependent expression of the Cyp2b10 and UGT1A1 genes in murine liver by exposure of mice to phenethyl isothiocyanate is mediated by ROS [32]. However, the notion of a ROS-mediated effect of the isothiocyanate on CAR is based solely on the use of the thiol N-acetyl cysteine, a rather unspecific and indirect modulator of ROS levels and obviously also a modulator of thiol/disulfide equilibria in proteins.

In summary, xenobiotics may affect FOXO activity through stimulation of CAR and/or PXR. Owing to their interaction with FOXOs, CAR and PXR are not only regulators of xenobiotic metabolism but also play an important role in the modulation of energy metabolism [33].

### 3.2 PPARs

Peroxisome proliferator-activated receptors (PPARs) represent a family of transcription factors that can be activated by diverse endogenous and exogenous ligands (such as the compounds originally categorized as “peroxisome [or microbody] proliferators”, i.e. compounds triggering peroxisome production [34]), rendering them targets susceptible to pharmacological and environmental xenobiotics and/or their cellular metabolites. The three PPAR proteins PPARα, PPARβ/δ and PPARγ exert isoform- and cell type-specific functions in the regulation of nutrient homeostasis and energy balance with emphasis on lipid and carbohydrate metabolism. PPARα and PPARγ show tissue-specific expression patterns: PPARα is enriched in metabolically active tissues such as liver, heart, kidney and intestine, while PPARγ is most highly expressed in mature adipocytes of the white adipose tissue. PPARβ/δ is ubiquitously expressed. Similar to CAR and PXR, active (ligand-bound) PPARs form heterodimers with retinoid X receptor (RXR) and bind to defined consensus sequences, PPAR response elements (PPREs), in the promoters of their target genes [35]. It is not fully established yet to what extent ligand binding supports nuclear localization of PPARs ([36] see Fig. 3**c**).

**Fig. 4:**
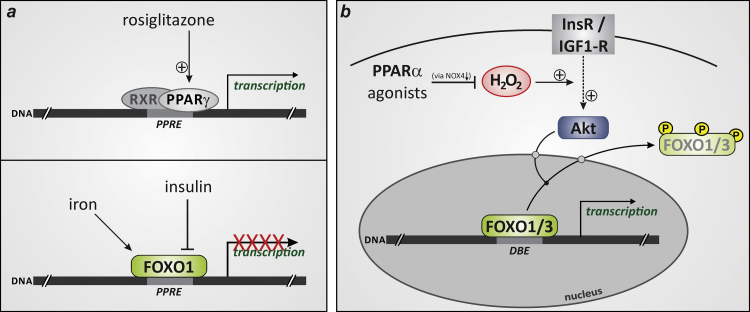
PPARs and FOXO transcription factors. (**a**) Binding of PPARγ to PPRE (peroxisome proliferator response element) sites is increased by agonists such as rosiglitazone [35], [38] (upper panel). Binding of FOXO1 at PPREs blocks transcription of PPARγ target genes. Iron augments this transrepressor activity of FOXO1 [40], whereas insulin induces inhibition and nuclear exclusion of FOXO1 [37], [38] (lower panel). (**b**) PPARα agonists enhance FOXO1/3 transcriptional activity through downregulation of NOX4 [42]. Downregulation of NOX4 means that less hydrogen peroxide is generated. As H_2_O_2_ is a known contributor to insulin signaling [43], this implies that less Akt activation as well as Akt-dependent FOXO inactivation will occur.

FOXO1, the most abundant FOXO isoform in adipose tissue, has been found to interfere with PPARγ-controlled gene expression in mature adipocytes in two ways (Fig. 4**a**): (i) FOXO1 may repress the promoters, and thus impair transcription of, the PPARγ1 and PPARγ2 genes [37]; and (ii) FOXO1 may directly bind to PPREs in promoters of PPARγ target genes, thus acting as a trans-repressor of PPARγ [38]. Binding of FOXO1 to PPREs is not mediated by its N-terminal DNA binding domain [38] that is otherwise required for interaction of FOXO1 with FOXO-responsive (DBE) sites within promoters of its proper target genes [5]. Instead, FOXO1 binds to PPREs through a centrally located and evolutionarily conserved 31 amino acid-domain containing an LXXLL motif [38].

The FOXO1-PPARγ interaction has been shown to be influenced by factors that control differentiation of preadipocytes into adipocytes as well as metabolic and endocrine functions of mature adipocytes (Fig. 4**a**): by inducing phosphorylation and subsequent nuclear exclusion of FOXO1, insulin counteracted FOXO1-mediated repression of PPARγ1 and PPARγ2 promoters as well as FOXO1 occupancy at PPRE sites in the promoters of PPARγ target genes, which in turn enhanced PPARγ transcriptional activity [37], [38]. This has been considered as a feed-forward mechanism contributing to maintaining insulin responsiveness of adipocytes [37], [38]. Insulin also rescued the induction of PPARγ target genes in FOXO1-overexpressing adipocytes exposed to rosiglitazone [38]. The anti-diabetic thiazolidinedione derivative rosiglitazone is a pharmacological PPARγ agonist that promotes adipocyte differentiation and lipid accumulation [35]. FOXO1 overexpression was capable of dampening but not of completely suppressing the rosiglitazone-induced increase in PPARγ transcriptional activity in adipocytes [38]. In this regard, it would be of interest to explore the impact of environmental PPARγ-targeting obesogens (adipocyte differentiation and lipid accumulation-promoting xenobiotics) such as tributyltin or phthalates [39] on the FOXO1-PPARγ interaction. Additionally, other substances derived from environment or nutrition may affect binding of FOXO1 to PPRE sites in PPARγ target genes, as demonstrated for the essential trace element iron: exposure of adipocytes to iron ions inhibited transcription and secretion of the PPARγ target gene adiponectin, an insulin-sensitizing adipokine, through induction of FOXO1 binding to a PPRE site in the adiponectin promoter [40].

The above-discussed actions of FOXO1 as a transcriptional trans-repressor appear to be restricted to PPARγ, as FOXO1 overexpression neither inhibited PPARα nor PPARβ/δ transcriptional activity [38]. However, a more indirect link was reported for FOXOs (FOXO1 and FOXO3) and PPARα in kidney (Fig. 4**b**). Two groups found that up-regulation/activation of PPARα was accompanied by enhanced FOXO transcriptional activity due to inhibition of Akt-mediated FOXO phosphorylation [41], [42]. Treatment with the pharmacological PPARα agonist, fenofibrate, protected spontaneously hypertensive rats from renal lipid accumulation and apoptotic cell death induced by feeding a high-fat diet [41]. Concomitantly, Akt and FOXO3 phosphorylation was suppressed in the kidneys of fenofibrate-treated animals, resulting in increased gene expression of the FOXO target genes SOD2, an antioxidant enzyme, and Bcl-2, an anti-apoptotic protein [41]. A recent study shed light on the molecular mechanism underlying the closely linked PPARα and FOXO activation by applying another pharmacological PPARα agonist, 2-[4-(5-chlorobenzothiazothiazol-2-yl)phenoxy]-2-methyl-propionic acid (MHY908). Similar to fenofibrate, MHY908 suppressed basal as well as insulin-induced Akt and FOXO1 phosphorylation in the kidneys of aged rats and in HEK293T human embryonic kidney cells, respectively; FOXO3 activation resulted in up-regulation of two FOXO-dependent antioxidant enzymes, SOD2 and catalase [42]. Use of PPARα-specific siRNA revealed that MHY908, through activation of PPARα, suppressed the insulin-induced and NADPH oxidase (Nox) 4-mediated increase in intracellular hydrogen peroxide levels in HEK293T cells [42]. Downregulation of Nox4 expression is likely to explain the activation of FOXO signaling by PPARα agonists, as the insulin signaling cascade that promotes phosphorylation (inactivation) of FOXOs is known to be enhanced by Nox4-mediated generation of H_2_O_2_ [5], [43].

### 3.3 Arylhydrocarbon receptor (AhR)

The arylhydrocarbon receptor (AhR, Fig. 3**d**) is a member of the bHLH-PAS family [with a basic helix-loop-helix DNA binding domain and Per-ARNT-Sim (PAS) domains] that is relatively ubiquitously expressed in human tissues (albeit to varying extents). Stimulation of AhR, which is kept in the cytosol by binding partners (including Hsp90 as well as AhR interacting protein, AIP), is achieved through binding of a ligand and the subsequent release of AhR binding partners, followed by nuclear translocation. AhR then heterodimerizes with ARNT (AhR nuclear translocator) and binds to DNA at specific sites, the xenobiotic response elements (XRE). Classical ligands promoting AhR activation include TCDD (2,3,7,8-tetrachlorodibenzo-*p*-dioxin) and other halogenated aromatic hydrocarbons, polycyclic aromatic hydrocarbons (PAH, such as 3-methylcholanthrene or benzo(a)pyrene), as well as indole derivatives and certain flavonoids. Target genes include CYPs, predominantly CYPs 1A1, 1A2, 1B1, as well as genes coding for phase II enzymes (e.g., UGTs) [25], [44].

**Fig. 5:**
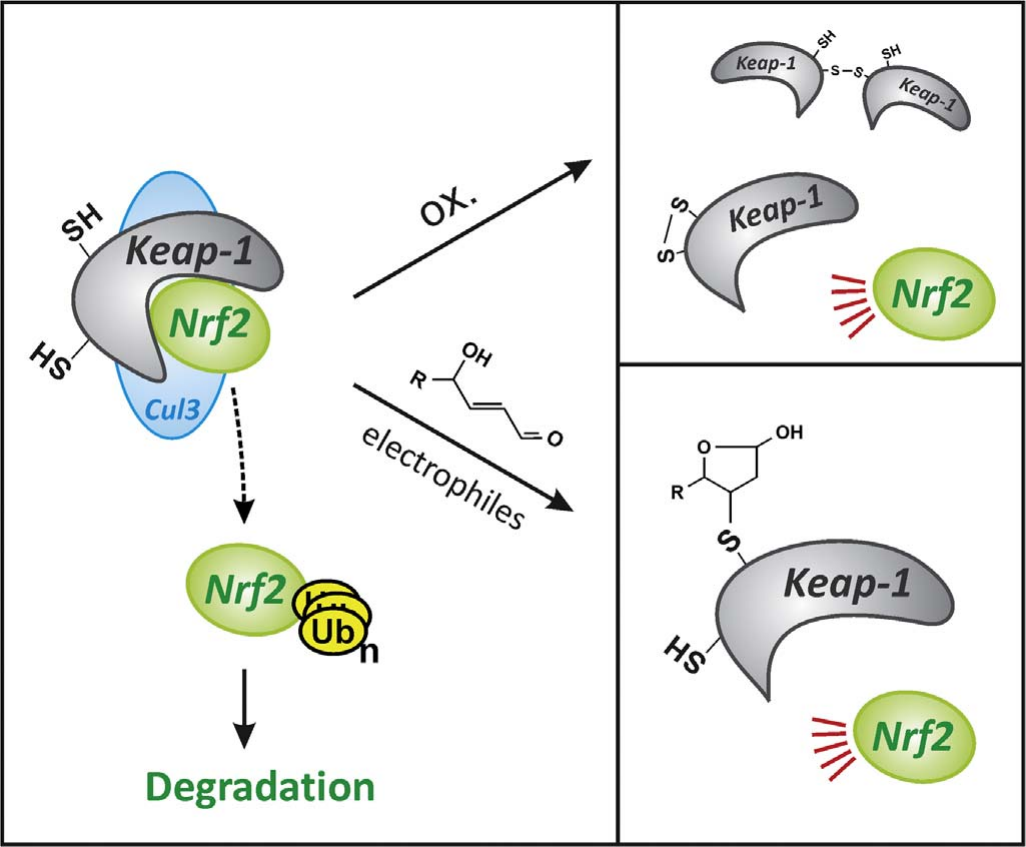
Nrf2 activation by xenobiotics. Xenobiotics may stimulate the transcriptional activity of Nrf2 through the generation of ROS or via the formation of electrophiles. Nrf2 is controlled by binding to Keap-1, which bridges Nrf2 and Cullin-3 (Cul3), a ubiquitin ligase, thus initiating proteasomal degradation of Nrf2 and preventing its nuclear translocation. Oxidation of Keap-1 (upper right panel) by ROS may induce disulfide formation (both intra- and intermolecular) [71], causing the release of Nrf2 from the complex, triggering Nrf2 nuclear translocation and transcriptional activation of target genes. Electrophiles may form adducts with Keap-1 through cysteine residues to elicit Nrf2 release and activation (lower right panel). The figure shows the adduct of Keap-1 generated upon reaction with the lipid peroxidation product, 4-hydroxynonenal (HNE) [72]; different electrophiles may react with different Keap-1 cysteines, as was demonstrated for compounds such as diethyl maleate and HNE [73], [74].

AhR interacts with a major regulator of the cellular antioxidant response, Nrf2 (see 3.4.); as summarized in a recent review [45], this occurs (i) via stimulation of CYP1A1-dependent metabolism of xenobiotics that results in the generation of ROS, which in turn stimulate Nrf2 (**see** Fig. 5), (ii) via transcriptional stimulation of Nrf2 synthesis by AhR, and (iii) by cooperation of AhR and Nrf2 in regulating antioxidant proteins such as NAD(P)H:quinone oxidoreductase-1 (NQO1; see Fig. 1**c**). A link between AhR and FOXO-dependent signaling was suggested in preliminary studies on FOXO-responsive promoter constructs that were activated by AhR agonists and then demonstrated to contain an artificially inserted XRE; deletion of this XRE rendered these FOXO-responsive luciferase constructs refractory to FOXO1-induced stimulation, suggesting that XRE and FOXO-responsive elements might interact [46]. Moreover, AhR was demonstrated to drive transcription of the CAR gene [47], enhancing CAR synthesis. As CAR is a corepressor of FOXO activity (see above), this would establish a link between AhR and FOXOs. PXR, which interacts with FOXO1 (see above), also interacts with AhR, yet it suppresses AhR transcriptional activity, apparently preventing AhR binding to XRE [48].

In summary, CAR/PXR, PPARs and AhR are closely linked in regulating xenobiotic metabolism, and redox regulation may occur at the level of their interaction with redox-regulated coregulators or transcription factors, such as FOXOs or nuclear factor erythroid 2-related factor 2 (Nrf2).

### 3.4 Nrf2

Nrf2 is a transcription factor of the cap’n’collar basic region leucine zipper (CNC-bZIP) family [49] stimulated by xenobiotics through suppression of its negative regulator, Keap-1 (Kelch-like ECH-associated protein 1). The latter constitutively mediates ubiquitination (and degradation) of Nrf2, thus keeping Nrf2 transcriptional activity under tight control (Fig. 5). Xenobiotics that affect Keap-1/Nrf2 interaction, leading to Nrf2 activation, are usually compounds interacting with thiols, such as (i) electrophiles capable of modifying one of the Keap-1 cysteine residues, (ii) compounds metabolized to such electrophiles, (iii) certain metal(loid) ions or (iv) compounds that cause the cellular generation of reactive oxygen or nitrogen species. Such reactive species will then directly oxidize Keap-1 cysteines or contribute to the cellular generation of electrophiles, such as 4-hydroxynonenal (HNE), a lipid peroxidation product (Fig. 5). Exposure to HNE causes stimulation of Nrf2 transcriptional activity in PC12 cells, followed by an enhanced cellular resistance against subsequent stress [50] – which is plausible considering the known Nrf2 target genes that encode phase II enzymes (such as GSTs, UGTs), but also proteins involved in antioxidant response, such as NQO1 (see Fig. 1c) or GCS1 (γ-glutamylcysteine synthetase, also known as GCL, glutamate-cysteine ligase), the rate-limiting enzyme in glutathione biosynthesis [49].

PI3K/Akt was shown to be involved in this response to HNE [50], pointing to the known link between receptor tyrosine kinase-dependent signaling, Akt (which is redox-regulated itself, see Fig. 1) and Nrf2, likely through inhibitory phosphorylation of glycogen synthase kinase (GSK) 3 [49], [51].

**Fig. 6:**
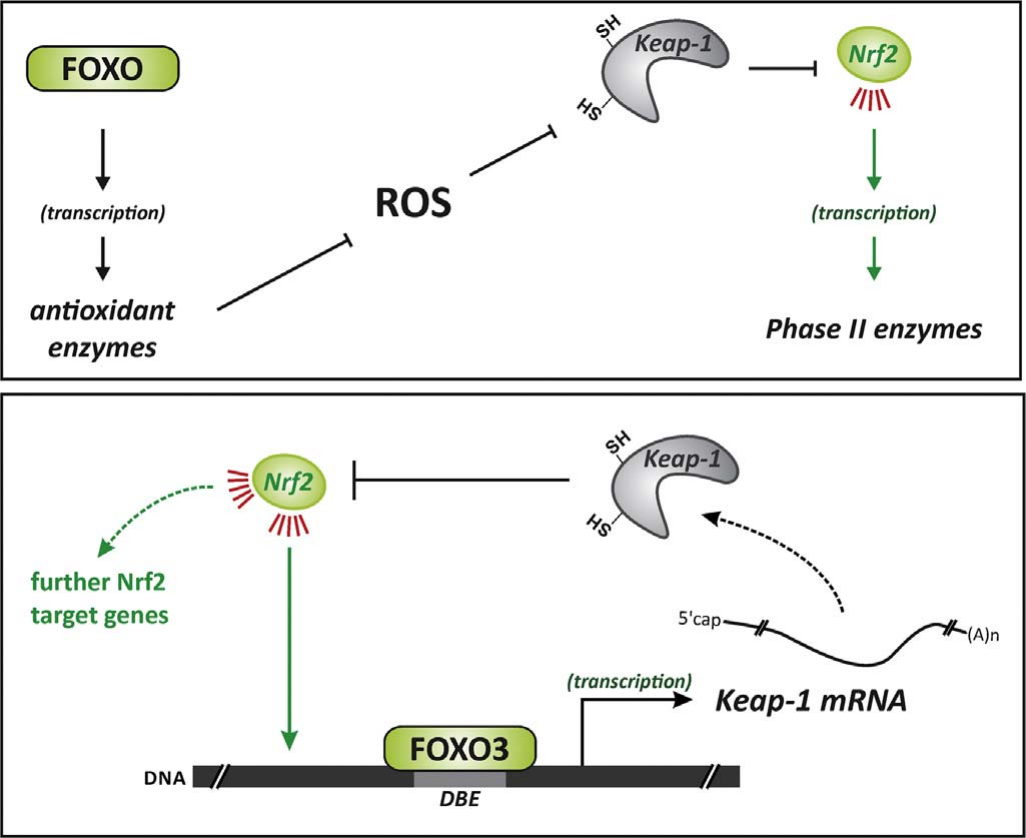
Links between Nrf2 and FOXOs. Upper panel: reactive oxygen species (ROS), e.g. by interacting with Keap-1, trigger Nrf2 activation. As FOXO proteins control the expression of genes coding for antioxidant proteins, the activation of FOXO may, by blunting surges in levels of ROS, ameliorate the activity of Nrf2. Lower panel: FOXO3 was shown to also attenuate Nrf2 activity by transcriptionally upregulating the biosynthesis of Keap-1 [52], which will bind and control Nrf2.

It was demonstrated in several human tumor cell lines cells that FOXO3 (but interestingly neither FOXO1 nor FOXO4) stimulates the transcription of the Keap-1 gene [52], regulating Keap-1 protein levels (Fig. 6); of note, this FOXO3/Keap axis appears to exist in human but not murine cells [52]. FOXO3 would, therefore, attenuate Nrf2 action by elevating Keap-1 levels, whereas active Akt would tend to stimulate Nrf2 by blocking said FOXO activity. This is in line with the positive action of Akt on Nrf2 activity described above. An additional link between the two transcription factor systems was hypothesized: xenobiotic-induced FOXO3 formation may require Nrf2, as shown in murine ovaries exposed to 4-vinylcyclohexene diepoxide [53].

A similar link was seen in *Caenorhabditis elegans* exposed to an electrophilic thiol depleting compound, diethyl maleate (DEM). Whereas DEM lowered *C. elegans* lifespan at higher concentrations, exposure of the nematodes to 10–100-fold lower concentrations enhanced their stress resistance and lifespan. This extension of lifespan was then demonstrated to require both DAF-16 (the FOXO ortholog in *C. elegans*) and SKN-1 (the *C. elegans* ortholog of Nrf2) [54]. The nature of their cooperation remains to be explored.

## 4 Conclusions

**Fig. 7:**
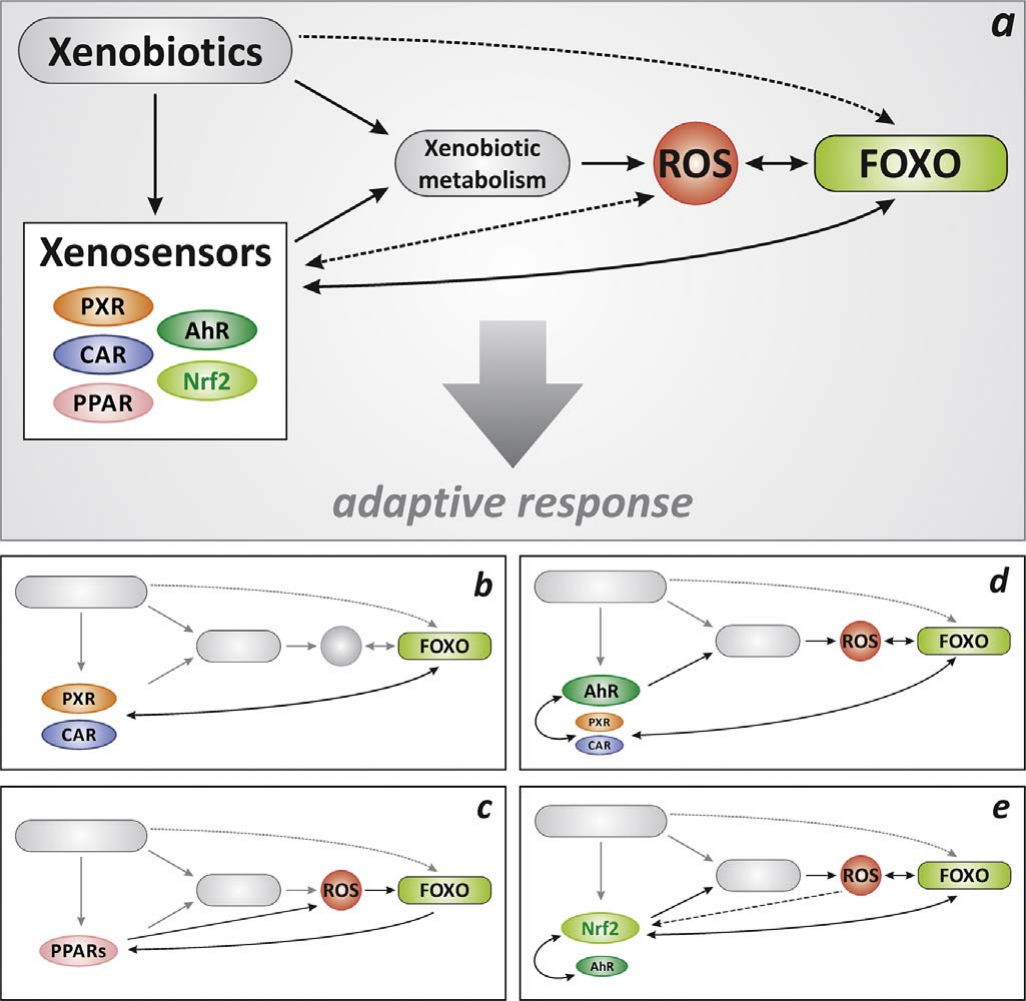
Cellular adaptation to an exposure to xenobiotics: contributions of xenosensors, FOXOs and ROS. (**a**) Xenobiotics, in part through the generation of ROS via xenobiotic metabolism, influence FOXO activity. In parallel, xenosensor/xenobiotic interaction will affect xenobiotic metabolism. FOXOs, in turn, act back on xenosensors in a variety of ways: the interactions of FOXOs with individual xenosensors explained in the respective chapters is briefly summarized in panels (**b–e**).

Exposure of cells to xenobiotics comes with an activation of xenosensors, such as nuclear receptor xenosensors (e.g., CAR, PXR, PPARs), AhR or Nrf2. It is also frequently accompanied by the generation of ROS, which may, in turn, affect xenosensor activities. Here, we have summarized literature data that lead us to conclude that FOXO transcription factors interact with all of the described xenosensors (Fig. 7). In some cases, this is a direct physical interaction with functional consequences, as in the case of CAR and PXR (Fig. 7**b**). In others (Fig. 7**c–e**), the interaction is indirect, either established through ROS (see PPARα, which may downregulate cellular H_2_O_2_ generation and thus modulate FOXOs, Fig. 4**b**; or see Nrf2, Fig. 6), through a competition for DNA binding sites (e.g., Fig. 4**a**) or through the mutual influence established by proteins whose biosynthesis is under the transcriptional control of either FOXOs or xenosensors (e.g., Fig. 6).

Altogether, these findings establish a picture that suggests a regulatory or modulating role of FOXOs and ROS regarding xenosensor activities. FOXOs, as redox-regulated transcription factors [5], may bridge xenobiotic-induced generation of ROS and the modulation of xenosensor activities. Finally, there are some consequences that point beyond this fine-tuning role of FOXOs and ROS:

(1) The interaction of xenosensors with FOXOs opens up the range of biological consequences of xenosensor activation: for example, CAR and PXR affect energy metabolism through their interaction with FOXOs [33]. Further activities (antioxidant response, autophagy, DNA repair etc) that FOXOs might have when under the influence of xenosensors remain to be explored.
(2) The role of ROS in the interaction maps emerging from the summarized findings (Fig. 7) suggests that xenosensor activities may be modulated by several conditions and stimuli that come with the generation of ROS, even independently of an exposure to a xenobiotic. Such conditions may be physical stimuli, such as ultraviolet radiation, which is a known trigger for the formation of singlet oxygen, superoxide and hydrogen peroxide in cells [55], or any dysregulation of ROS generating enzymes.
